# Forensic science: defending justice

**DOI:** 10.1080/20961790.2016.1243083

**Published:** 2016-12-12

**Authors:** Min Shen, Duarte Nuno Vieira

**Affiliations:** Shanghai Key Laboratory of Forensic Medicine,Shanghai Forensic Service Platform, Institute of Forensic Science, Ministry of Justice, PRC, Shanghai, China shenm@ssfjd.cn; Faculty of Medicine, University of Coimbra, Coimbra, Portugal

Forensic science is the application of scientific knowledge and methodology for the resolution of legal questions and problems for individuals and society. It involves the observation, documentation, collection, analysis, assessment and scientific interpretation of evidence during the course of an investigation required for the different fields of law, including criminal, civil, work, family and administrative. Forensic scientists also testify as expert witnesses and can work for either the prosecution or the defence.

Over the past few years, an abundance of new insights and technologies has caused the forensic science field to grow rapidly. Moreover, advances in technology mean that forensic scientists are able to do much more with the same resources than they were before, so the value of the forensic laboratory has increased significantly.

The role of a scientific journal is to disseminate science, to be a tool for international research communication. Today there are only 15 Science Citation Index (SCI) forensic science journals in the world, and their impact factors are at a much lower level than journals in other disciplines. All 15 of these SCI journals are almost monopolized by European and North American publications, with few Asian journals indexed by the SCI. More and more scientific achievements in forensic science are emerging, and forensic scientists face the challenge of increasing the quantity and quality of their research. These journals cannot meet all the needs of these developments. The range of journals available is narrower for forensic science than for many other fields. To meet this demand and further serve the needs of academia, industry and policy analysis, we are pleased to announce the launch of a new global and multidisciplinary journal, called *Forensic Sciences Research* (*FSR*).

The Institute of Forensic Science (IFS) attached to Ministry of Justice, P.R. China, will be the sponsor of *FSR*. It is the oldest full-scale institute specializing in multi-disciplinary forensic scholarship, with nearly 100 years of history in China. The IFS is very active in international scholarship exchanges and has developed international training programmes with many forensic institutes across the world. The IFS will provide a valuable foundation for the successful development of *FSR*. China has a long history of forensic science. The first written account of using medicine and entomology to solve criminal cases is attributed to the book of *Xi Yuan Ji Lu* (translated as *The Washing Away of Wrongs*), which was written in Chinese by Song Ci (1186–1249) in 1248, during the Song Dynasty. The book offered advice on how to determine a drowning case (water in the lungs) or strangulation (broken neck cartilage), along with using other evidence from corpses to distinguish deaths caused by murder, suicide or accident. Over the past 30 years, there has been an extremely rapid development of the Chinese economy, and the Chinese government now invests a large amount of funding to scientific research, technology and education every year. The number of research papers published in China has significantly increased to be second only to United States in the past few years. Many of these research papers have a high citation rate by well-regarded academic journals. Additionally, China has a strong ethos of promoting academic communication through scholarly publication. As early as the 1930s, Professor Lin Ji, the founder of modern Chinese forensic science, founded the Journal of *DIE GERICHTLICH-MEDIZINISCHE MONATSSCHRIFT* in Shanghai.

In the subsequent decades, Chinese forensic scientists have shared and communicated a multitude of scholarly works with researchers and scholars worldwide, through major academic journals such as *Forensic Science International*, *Journal of Forensic Sciences*, *International Journal of Legal Medicine*, *Science & Justice and Forensic Science, Medicine, and Pathology*. Many other Chinese papers of high quality have also been published in various fields of forensic medicine and science including forensic pathology, clinical forensic medicine, forensic psychiatry, forensic toxicology, forensic biology, forensic genetics, anthropology, document examination, forensic entomology and forensic odontology, to effectively promote the application of their research outcomes. Ultimately, Chinese scholars make strong contributions to forensic science internationally. According to Web of Science, Chinese papers published in these 15 SCI journals increase every year, reaching 297 in 2014 and 2015.

*FSR* will be published as a peer-reviewed journal, sponsored by the IFS with the cooperation of the Taylor & Francis Group in England. Initially it will be a quarterly periodical publication in English. The mission of this journal is to offer an academic platform for forensic scientists and researchers to publish and exchange interesting, challenging and innovative research findings across various disciplines related to collecting, preserving and analysing scientific evidence during the course of a forensic investigation. We hope that *FSR* will provide an effective communication platform for all forensic scholars in this era of globalization, and serve as a role model for other Chinese forensic science journals to follow in the future.

*FSR* welcomes forensic science and technology manuscripts in all forensic sciences areas, namely in forensic pathology, clinical forensic medicine, forensic psychiatry, forensic toxicology and chemistry, forensic biology, forensic genetics, anthropology, criminalistics, document examination, accident investigation, crime scene investigation, explosives, quality assurance, forensic entomology, forensic odontology, digital & media sciences, gunshot injury and engineering sciences, etc., as well as investigations of value to public health in its broadest sense, and the important cross-disciplinary areas where science and medicine interact with the law. *FSR* is intended for publishing original research, scholarly reviews, opinion papers and research highlights/commentaries in forensic science. Through these features, *FSR* aims to build a communication platform for international researchers to share scholarly achievements effectively. In addition to full scientific research or technological and application papers, shorter papers of academic interest, reports on conferences and reviews of books in relevant fields are also welcome.

*FSR* has adopted internationally acknowledged standards for and approaches to its operation. The journal is governed by a highly esteemed academic editorial board consisting of internationally recognized world-class scholars from different countries, including the United States, the United Kingdom, Germany, France, Australia and China. The Board membership has met the high academic standards required for an international scholarly journal for submission, peer-review (*FSR* has established an editorial manager) and publication (production and hosting by the Taylor & Francis Group).

After a year of preparatory work, *FSR* is now ready to be launched. On this special occasion, we would like to express our sincere thanks to all those authors that gave their contribution for this first issue. Thanks also go to all members of the Editorial Board of *FSR*, our publisher Taylor & Francis Group and expert reviewers who have made significant contributions to the publication of this journal. It is only with their support that the editorial team has been able to put together this inaugural issue.

We hope that *FSR* is successful in serving as an international academic forum for scholars to communicate and exchange their ideas, research approaches, results and experiences. *FSR* should become the journal of choice for international forensic practitioners and scholars to share and advance knowledge and to learn from each other across the forensic sciences. We welcome your comments on this first issue and suggestions for future ones, as well as on any other aspect of the *FSR*. It is our goal to work together for *FSR* to uphold high standards, integrity and excellence in its publication. Your support will be a strong driving force for us in our efforts to continually improve the quality and influence of this journal.

